# Supplementary figures and images for: A novel mean shape based post-processing method for enhancing deep learning lower-limb muscle segmentation accuracy
Source: PLoS One. 2024 Oct 4;19(10):e0308664. doi: 10.1371/journal.pone.0308664 (PMC11452003; doi:10.1371/journal.pone.0308664)

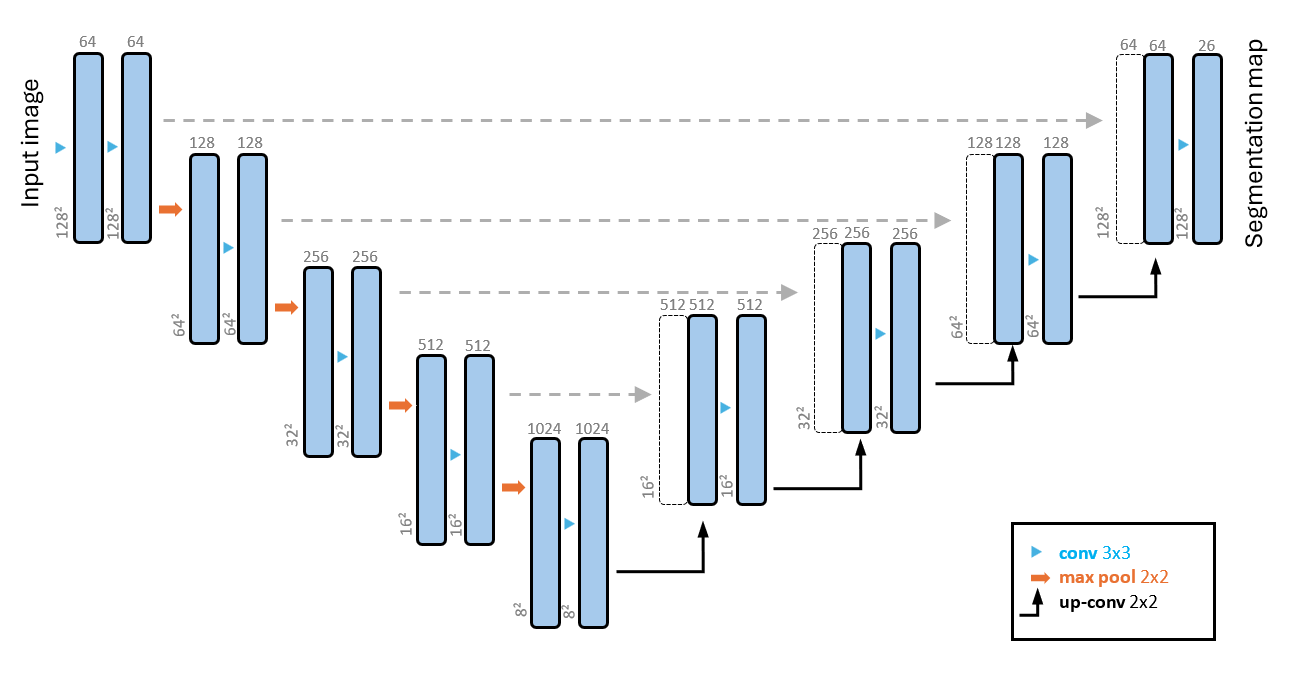

Supplement: S1 Fig — (TIFF) [file pone.0308664.s001.tiff]
