# Supplementary material for: A novel mean shape based post-processing method for enhancing deep learning lower-limb muscle segmentation accuracy
Source: PLoS One. 2024 Oct 4;19(10):e0308664. doi: 10.1371/journal.pone.0308664 (PMC11452003; doi:10.1371/journal.pone.0308664)
Supplement: S2 Fig — Subplots (s1-s8) represent the performance of four metrics (DSC/RVE/HD/ASSD) for each subject (8 in total) under different methods (each boxplot includes 16 individual muscles’ score). The median number of each boxplot is connected by dotted red lines to see if the overall trend is the same. (PDF) [file pone.0308664.s002.pdf]

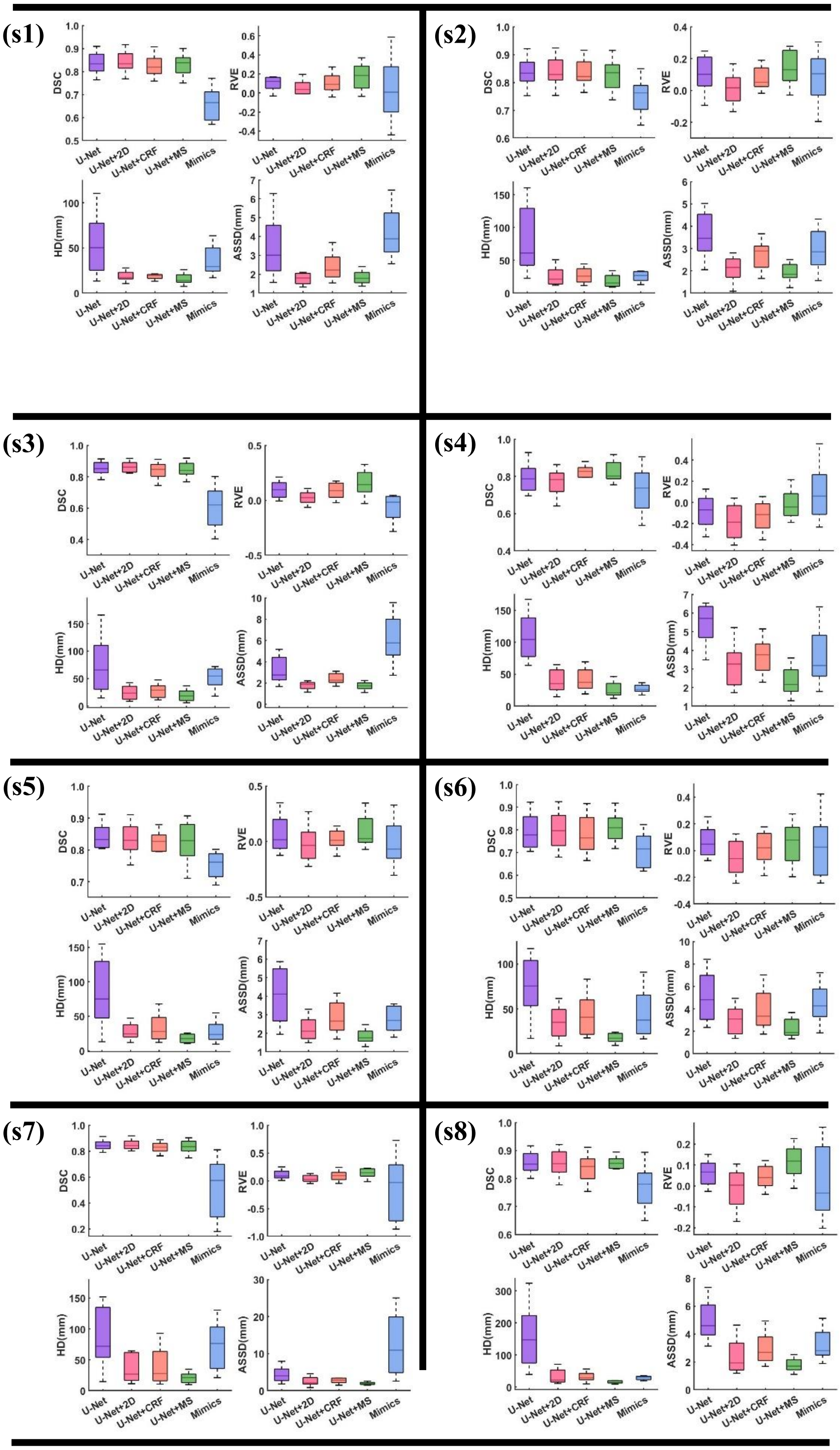

S2 Fig Performance comparison of each method on individual testing subjects from PMW-2. Subplots (s1-s8) represent the performance of four metrics (DSC/RVE/HD/ASSD) for each subject (8 in total) under different

methods (each boxplot includes 16 individual muscles' score). The median number of each boxplot is connected by dotted red lines to see if the overall trend is the same.
