# Supplementary material for: A novel mean shape based post-processing method for enhancing deep learning lower-limb muscle segmentation accuracy
Source: PLoS One. 2024 Oct 4;19(10):e0308664. doi: 10.1371/journal.pone.0308664 (PMC11452003; doi:10.1371/journal.pone.0308664)
Supplement: S1 Table — The volume of each muscle in the table is achieved by averaging the corresponding muscle volumes across all subjects. There is no statistically significant difference in muscle volume between the two cohort. (DOCX) [file pone.0308664.s003.docx]

| **Muscle** | **PMW-1 [cm^3^]** | **PMW-2 [cm^3^]** |
| --- | --- | --- |
| Rectus femoris | 127 | 151 |
| Vastus intermedius | 294 | 277 |
| Vastus lateralis | 376 | 422 |
| Vastus medialis | 247 | 281 |
| Sartorius | 108 | 110 |
| Semimembranosus | 136 | 158 |
| Semitendinosus | 119 | 117 |
| Gracilis | 60 | 67 |
| Biceps femoris caput brevis | 68 | 63 |
| Biceps femoris caput longum | 127 | 145 |
| Adductor magnus | 390 | 366 |
| Adductor brevis | 70 | 62 |
| Adductor longus | 90 | 95 |
| Gluteus maximus | 643 | 641 |
| Iliacus | 136 | 147 |
| Tensor fasciae latae | 48 | 51 |
| $\boldsymbol{p>0.05}$ | | |

**S1 Table. Muscle average volume comparison between PMW-1 and PMW-2.**

The volume of each muscle in the table is achieved by averaging the corresponding muscle volumes across all subjects. There is no statistically significant difference in muscle volume between the two cohort.
