# Supplementary material for: A novel mean shape based post-processing method for enhancing deep learning lower-limb muscle segmentation accuracy
Source: PLoS One. 2024 Oct 4;19(10):e0308664. doi: 10.1371/journal.pone.0308664 (PMC11452003; doi:10.1371/journal.pone.0308664)
Supplement: Attachment — Submitted filename: Review.docx [file pone.0308664.s004.docx]

This study introduces a new post-processing method that compensates for the shortcomings of UNet-based segmentation methods based on Magnetic Resonance Imaging (MRI) scans and verifies its effectiveness in practice. The proposed method takes into consideration the muscle anatomical shape, enabling more effective and accurate segmentation of muscles. I believe that it is useful as it can be applied to other organ segmentation such as bone, lung etc. By comparing with existing post-processing approaches as well as a commercial segmentation software, it has demonstrated the good performance of the proposed post-processing approach. The paper is very well organised and written. Comments are:

1. The contribution of the study should be highlighted.
2. Obviously, the proposed post-processing approach can be apply in any segmented images. There are many different types of deep learning networks, can you explain why UNet was chosen as the deep learning model for this study?
3. In Participants, it looks like you used two different MRI scanners, were there any differences in imaging conditions between the two cohorts?
4. For the operators for manual segmentation, were they the same people or different people with similar technical level?
5. For the training, one cohort of data (PMW-1) was used as training data while the second cohort was used for test. Is there any population bias in the cohort which may affect your result?
6. In the proposed method, mean shape of the cohort (postmenopausal women) was used. What is your view for the method to apply to different population, say young people?
