# Supplementary material for: A novel mean shape based post-processing method for enhancing deep learning lower-limb muscle segmentation accuracy
Source: PLoS One. 2024 Oct 4;19(10):e0308664. doi: 10.1371/journal.pone.0308664 (PMC11452003; doi:10.1371/journal.pone.0308664)
Supplement: Attachment — Submitted filename: Response to Reviewers.docx [file pone.0308664.s005.docx]

**Reviewer 1 major comments.**

1)

**Q) The contribution of the study should be highlighted.**

A) Thank you for your suggestions. The contribution of the study has been added in the end of the introduction section.

**note)** From Line 99 to 103, the contribution of this study has been highlighted: ‘The contributions of this study are: 1) A new mean shape (MS) based post-processing method is proposed, utilizing Statistical Shape Modelling (SSM) to fine-tune the segmentation output, taking into consideration the muscle anatomical shape; 2) A comparative study is conducted to investigate the effectiveness  of  post-processing approaches to  the improvement of the DL automatic segmentation accuracy in terms of DSC, HD and ASSD.’

2)

**Q) Obviously, the proposed post-processing approach can be applied in any segmented images. There are many different types of deep learning networks, can you explain why UNet was chosen as the deep learning model for this study?**

A) The primary objective of this study is to investigate whether the proposed mean shape based post-processing method improves segmentation results. To achieve this, as a widely used segmentation model, UNet was chosen for training. Compared to other advanced models, UNet has fewer parameters, ensuring it does not excessively consume computational resources. Additionally, if the post-processing method proposed in this study proves to be effective, it can then be easily and directly applied to the output results of other studies that use UNet for training. This will facilitate direct comparisons between the original results and those obtained using our method.

3)

**Q) In Participants, it looks like you used two different MRI scanners, were there any differences in imaging conditions between the two cohorts?**

A) Although different scanners are used, the parameters used for imaging are the same. The imaging and post-processing operation details have been added in the revised draft.

**note)** From Line 112 to 123: ‘Although the two cohorts were imaged in separate studies, the scans were conducted at the same hospital using two different 1.5T MRI scanners, following the same scanning protocol. All complete lower-limb MRI scans were performed using the 1.5T Siemens Magnetom Avanto or the 1.5T Siemens Magnetom Aera (Siemens AG, Erlangen, Germany). Four sequences were acquired to image the hip, thigh, knee, and shank [21]. To reduce scanning time without compromising the detailed geometry of the joints, the joints were imaged at a higher resolution (pixel size of 1.05 mm × 1.05 mm and slice thickness of 3.00 mm) compared to the central portions of the long bones (pixel size of 1.15 mm × 1.15 mm and slice thickness of 5.00 mm). These sequences were then combined using MATLAB (R2006a) to create a continuous 3D image from the hip to the ankle. This was achieved by standardizing the resolution of each imaging sequence from the different sections through tri-linear interpolation (interp3, MATLAB R2006a) to 1.00 mm × 1.00 mm × 1.00 mm.’

4)

**Q) For the operators for manual segmentation, were they the same people or different people with similar technical level?**

A) PMW-1 dataset was segmented by two PhD students and one postdoctoral researcher and PMW-2 dataset was segmented by one PhD student with similar technical level and trained on at least 15 datasets.

**note)** The sentence in section 2.1 from line 136 to 141 has been rephrased as follows: ‘The MR images of lower-limb muscles from PMW-1 were segmented by operators (two PhD students and one postdoctoral researcher) using the semi-automated muscle segmentation toolbox in Mimics 20.0 (Materialise, Leuven, Belgium), followed by some manual adjustments and post-processing [19]. The scans from PMW-2 were segmented by one operator (one PhD student) using the software Amira (version 2022.2; Thermofisher). All the operators have similar technical levels in terms of muscle segmentation and were trained on at least 15 datasets.’

5)

**Q) For the training, one cohort of data (PMW-1) was used as training data while the second cohort was used for test. Is there any population bias in the cohort which may affect your result?**

A) Both cohorts are composed of postmenopausal women, thus exhibiting similar physiological characteristics. As shown in Table 1, the average age difference is 2 years, and the average height is also similar. In comparative analysis, different methods are based on the results from the same samples (PMW-2), ensuring that the bias between PMW-1 and PMW-2 does not significantly impact this study's outcomes. Section 3.1 and the appendix of this paper provide a detailed analysis of the PMW-2 individuals (S2 Fig).

6)

**Q) In the proposed method, mean shape of the cohort (****postmenopausal women) was used. What is your view for the method to apply to different population, say young people?**

A) Compared to postmenopausal women, MRI muscle slices of younger individuals exhibit different textural characteristics. For example, due to ageing, muscle atrophy in postmenopausal women leads to less compact muscle boundaries (as shown in Fig. R1), facilitating better segmentation by DL models. In contrast, younger individuals have stronger muscles with potentially less distinguishable boundaries between adjacent muscles. Due to muscle loss with age, some soft tissues (such as fibers and ligaments) are relatively obvious, showing low signals on the T1 sequence and showing black textures in the images.Therefore, the output results of deep learning models are expected to vary across different cohorts.

Fig. R1 MRI slice comparison between different cohort

Regarding the proposed post-processing methods, this study focuses more on geometric morphological changes. If the pre-generated mean shape of a particular muscle closely resembles the shape and volume of the muscle in a new sample, the post-processing method would be effective. However, if there are significant differences in muscle volume, length, and morphology between the different cohorts, further research will consider generating different mean shape dataset for different cohorts to appropriately classify and process the incoming samples.

**Reviewer 2 major comments.**

1)

**Q) When data is pooled, is this done after the predictions? Or is a separate U-Net trained for the pooled data. If only 1 label is available for training the U-Net I imagine that you could achieve much better results compared to only pooling in post-processing.**
A) Yes, the pooling operation is done after the prediction. If a model is used to learn and predict a specific muscle (binary segmentation task), it is likely to yield more accurate results. However, this approach would require significantly more training time and computing consumption. In this study, we employ pooling to observe the differences in the recognition abilities of various methods on muscle regions at a macro level.

2)

**Q) Just reading the introduction, the goals of the study slightly confuse me. In L91 you mention that a new SSM-based post-processing method is proposed in this study. However, in the subsequent paragraph, you say that the goal of the study is to assess the effect of existing post-processing methods, with no mention of the proposed novel method. From the rest of the paper, it is clear that this SSM-based method is an essential part of the goal of this study.**

A) The sentence has been rephrased and added to introduction from line 95 to 103 as follows:

‘The goal of this study was to propose a new mean shape based post-processing method and to assess the effect of it with existing methods (CRF, and 2D post-processing method) on the accuracy of a previously developed CNN approach to segment individual muscles of the lower limb from MRI scans [4]. The secondary goal of the study was to compare the accuracy of different segmentation tools, including that of a commercial semi-automatic muscle segmentation toolbox (Mimics 26.0, Materialise). The contributions of this study are: 1) A new mean shape (MS) based post-processing method is proposed, utilizing Statistical Shape Modelling (SSM) to fine-tune the segmentation output, taking into consideration the muscle anatomical shape; 2) A comparative study is conducted to investigate the effectiveness of post-processing approaches to the improvement of the DL automatic segmentation accuracy in terms of DSC, HD and ASSD.’

3)

**Q) More information regarding the MR images should be presented (pixel size, slice thickness, slice increment, FOV, any filtering applied)**

A) The detailed information of the MR images has been added to the section 2.1 from line 112 to 123 as follows.

**note)** From Line 112 to 123: ‘Although the two cohorts were imaged in separate studies, the scans were conducted at the same hospital using two different 1.5T MRI scanners, following the same scanning protocol. All complete lower-limb MRI scans were performed using the 1.5T Siemens Magnetom Avanto or the 1.5T Siemens Magnetom Aera (Siemens AG, Erlangen, Germany). Four sequences were acquired to image the hip, thigh, knee, and shank [21]. To reduce scanning time without compromising the detailed geometry of the joints, the joints were imaged at a higher resolution (pixel size of 1.05 mm × 1.05 mm and slice thickness of 3.00 mm) compared to the central portions of the long bones (pixel size of 1.15 mm × 1.15 mm and slice thickness of 5.00 mm). These sequences were then combined using MATLAB (R2006a) to create a continuous 3D image from the hip to the ankle. This was achieved by standardizing the resolution of each imaging sequence from the different sections through tri-linear interpolation (interp3, MATLAB R2006a) to 1.00 mm × 1.00 mm × 1.00 mm.’

4)

**Q) L112-L124: it is unclear to me if the segmentations of PMW-1 were performed as part of this study, or in another study (reference 19). If I understand correctly, the scans for PMW-2 were segmented for this specific study. Were exactly the same muscles extracted? How was the segmentation performed? Was this also performed semi-automatically and performed by multiple operators and then compared? I believe that these segmentations are considered the golden standard mentioned in the results. I suggest mentioning this explicitly for clarity.**
A) Yes, PMW-1 was manually segmented in another study [ref 19], and PMW-2 was manually segmented in this study. The same muscles were extracted.

**note)** The sentence in section 2.1 has been rephrased from line 136 to 141 as follows:

‘The MR images of lower-limb muscles from PMW-1 were segmented by operators (two PhD students and one postdoctoral researcher) using the semi-automated muscle segmentation toolbox in Mimics 20.0 (Materialise, Leuven, Belgium), followed by some manual adjustments [19]. The scans from PMW-2 were segmented by one operator (one PhD student) using the software Amira (version 2022.2; Thermofisher). In the software, the operator manually selects the target region on each MR slice to perform purely manual segmentation. All the operators have similar technical levels in terms of muscle segmentation and were trained on at least 15 datasets.’

5)

**Q)** **I suggest trying to put table 2 into a flowchart figure as is often done with U-Net networks.**
A) Following the practice outlined in reference 6, the layers parameters of U-Net are described in more detail in table to facilitate comparison and can provide a reference for readers. Following the suggestion, a flowchart figure of the U-Net structure is added in supporting files (S1 Fig).

6)

**Q) L131: I'm a bit confused regarding how the U-Net was trained. Why was the U-Net trained and tested 3 times? Was it retrained for each method of post-processing? If so, wouldn't it make more sense to perform the post-processing on one trained model? Was there no validation set? What hyper-parameter settings were used?**

A) The purpose of the repeated training and testing is to provide a means for statistical analysis, which will be able to evaluate the effectiveness of the methods. In this study, we chose to repeat 3 times. Each time was followed by applying various post-processing methods to the results. The purpose of this study is not to select the best model architecture; therefore, no special validation set was reserved. During the training period, the dataset was randomly split into training and validation sets with a 9:1 ratio. The initial learning rate for the model was set to 0.001, and it decayed by 0.9 every 5 epochs, for a total of 100 epochs. The model was trained with the multi-class cross entropy loss function. Other hyper-parameters were tuned empirically for the optimal network performance, whilst ensuring the GPU memory and capacity were not exceeded. After the loss converged on the training set, the best-performing set of weights from each epoch on the validation set was retained.

**note)** The training detail information has been added to section 2.2 from line 147 to 152 as follows: ‘During the training period, the dataset was randomly split into training and validation sets with a 9:1 ratio. The initial learning rate for the model was set to 0.001, and it decayed by 0.9 every 5 epochs, for a total of 100 epochs. The model was trained with the multi-class cross entropy loss function. Other hyper-parameters were tuned empirically for the optimal network performance, whilst ensuring the GPU memory and capacity were not exceeded. After the loss converged on the training set, the best-performing set of weights from each epoch on the validation set was retained.’

7)

**Q) L141: I don't understand how muscles were 'manually selected using thresholding'.**
A) In the process of using Mimics, the software requires users to select the approximate region of the muscle in the MR slices as a mask without needing high precision. The thresholding method is one option provided by the software. Given the significant contrast between the muscle region and the surrounding tissues (e.g. fat tissue) in MRI slices, the thresholding method can conveniently select the approximate muscle area. During usage, the threshold is manually adjusted by observing the coverage of the muscle mask. The manual adjustment continues until the mask includes the approximate muscle region without covering excessive noise.

8)

**Q) Section 2.5, fig 3.: Why were the initial U-Net predictions used to build the SSM? I believe that it would be better to use the original gold standard semi-automatic segmentations would lead to more accurate mean shapes. Now you are already limited by how good the initial U-Net predictions are. If this is not a mistake in writing, I believe this is quite crucial and requires more explanation.**

A) The mean shape is derived from the precise manual segmentation results (gold standard) of PMW-1. This clarification has been further elaborated in the revised manuscript.

**note)** The sentence has been revised and added to section 2.3 from line 207 to 208 as below: ‘Using the ShapeWorks software, the mean shape for each muscle was generated (a-2) using gold standard manual segmentation from PMW-1.’

9)

**Q) L273: In other publications I have seen the RVE presented as an absolute value. Was it a conscious decision to not use the absolute volume difference?**
A) The reason for not using absolute values is to understand the specific differences in muscle volume prediction among different methods, whether they tend to overestimate or underestimate. If biases exhibit systematic consistency, adjustments can be made manually during practical application to align the results more closely with actual values.

10)

**Q) L326: I fail to see how the Hausdorff distance becomes meaningless with larger objects. Yes, the HD will likely increase compared to the analysis of the individual muscles, but it can still be used to compare the various post-processing methods, no?**
A)

*Original content: ‘ When the labels of the 16 target muscles were pooled into one label, DSC, RVE, and ASSD were used to evaluate the recognition performance of each method on the overall target muscle region (Fig 5). Because the whole muscle cohort is larger and longer than the individual muscle, the comparison of HD is meaningless and therefore removed.’*

For medical applications, measuring muscle volume is a focal point of researchers' attention. Pooling the 16 muscles’ region as a label increases the volume, where errors in the Hausdorff distance (HD) calculation may not significantly affect the overall volume as much as individual muscle volumes. Therefore, in this part, using Average Symmetric Surface Distance (ASSD) can better evaluate a model or method's robustness against noise compared to HD. ASSD calculates the average distance from each surface pixel to the gold standard shape's surface.

11)

**Q) Fig 7. I like this figure, but it's difficult to distinguish muscles where adjacent muscles are all colored dark blue.**
A) Thank you for the suggestion, the Fig 7 (as shown below) has been modified as required.

**Fig 7. Segmentation performance visualization of each method.**

12)

**Q) Figure 10 and table 4 present results and thus fit better in the results section.**
A) Many thanks for the suggestion. Figure 10 and Table 4 mainly discuss the comparison and difference of before and after using post-processing methods and suggestions on different methods chosen for different muscles in the future. The contents are obtained through discussion, so we think that it is more appropriate to show them in the discussion part.

13)

**Q) L460: 'General consistency between the cohorts' does not appear true to me. There may not be a statistical difference, but I can imagine that there is a difference in muscles between subjects with a BMI of 26.5 +- 3.4 and 22.0 +- 2.1. Did you check the difference in volume, shape, etc. between the muscles in PMW1 and PMW2?**

A) We examined the differences in muscle volumes between the two cohorts and found no statistically significant differences (S1 Table). Regarding BMI, most individuals' obesity is concentrated in the visceral area (abdominal region), thus, although there is a difference in average BMI, it is not a critical factor affecting muscle volume.

note) The following sentences have been rephrased to make the states clearer in conclusion part from line 486 to 487 as below: ‘The atlas files in this study were derived from PMW-1 cohort, while input samples were from PMW-2, with general consistency in age, volume ($p>0.05$, S1 Table), etc.’

14)

**Q) It would be interesting to read your opinion on the generalizability of the MS post-processing method. It appears to me that you're confronted with quite a lot of smoothing when using a mean shape and this doesn't change when adding more subjects. Does this make this method only applicable for healthy, near-average subjects?**

A) When adding more subjects, the obtained mean shape will more closely approximate the overall mean size and may be smoother or adding an opposite effect. Our post-processing method is suitable for individuals without significant geometric anomalies. In Fig. 9, we demonstrate that this method can effectively correct areas with cliff changes in muscle shape predictions and correct some notably erroneous predictions. Therefore, this method is confirmed to be effective for healthy individuals.

For non-healthy individuals, due to insufficient dataset, we did not test with special samples. However, we assume that if a disease affects the muscle such that the overall geometric shape remains unchanged but only the volume is affected, our method can still be effectively applied. In cases where diseases cause significant deformation of the muscle geometry, using the mean shape for post-processing might result in the predicted shape smoothing towards the healthy mean shape. The result may fall somewhere between the healthy and diseased shapes and may not be particularly smooth. Future research can further test the generalizability of our method by collecting data from such cases.

**Reviewer 3 major comments.**

1)

**Q) The paper is very well organised and written. English may be improved.**
A) Thank you for your suggestion. In the revision, we have corrected some grammatical errors and refined some expressions.

2)

**Q) You have compared your method with Mimics commercial semi-automatic muscle segmentation toolbox. What is the main purpose of this comparison? Does Mimic have post-processing?**
A)

Mimic does not have the function of post-processing. In ref 19, the researchers processed the samples by using Mimic first and then using manual operation to improve the segmentation results. We wanted to compare the method without post-processing to evaluate the potential of Mimics on its own. The study confirmed that post-processing would be needed.

3)

**Q) There are lots of deep learning algorithms for image segmentation, why UNet?**
A) The primary objective of this study is to investigate whether the proposed mean shape based post-processing method improves segmentation results. To achieve this, as a widely used segmentation model, UNet was chosen for training. Compared to other advanced models, UNet has fewer parameters, ensuring it does not excessively consume computational resources. Additionally, if the post-processing method proposed in this study prove to be effective, it can be easily and directly applied to the output results of other studies that use UNet for training. This will facilitate direct comparisons between the original results and those obtained using our method.

4)

**Q) In your comparison, Mimics has lower RVE, which is better than your method. Any consideration for future improvement of your algorithm?**
A) With more samples and further experiments, we can optimize the algorithm in terms of accurate volume prediction. Regarding the current suboptimal RVE performance, we can investigate whether there are systematic differences in specific muscles volume prediction. If such differences or gaps exist, further processing can be used to reduce the error. Alternatively, when new samples are input, we can estimate if there are significant volume differences between specific muscles by comparing the sample's average weight (which can be measured easily) with the cohort (which used to produce mean shape) in the database. This approach may allow for the introduction of correction factors to better adjust the predicted volumes.

5)

**Q) It looks like mean shape is the mean value of this specific cohort. What about other cohort?**

A) The effectiveness of the mean shape-based post-processing method proposed in this study primarily depends on the geometric similarity between the pre-processed mean shape from dataset and the new samples, as well as no significant volume differences. For different cohorts, if there are no significant gaps in muscle morphology and muscle volumes are within the same order of magnitude, this method remains applicable.

However, for groups with special conditions (where certain muscle shapes undergo unique changes) or groups with muscle volumes that differ significantly from the PMW dataset, future research will employ a classification-based mean shape database to process the prediction results.
